# Human macular Müller cells rely more on serine biosynthesis to combat oxidative stress than those from the periphery

**DOI:** 10.7554/eLife.43598

**Published:** 2019-04-30

**Authors:** Ting Zhang, Ling Zhu, Michele C Madigan, Wei Liu, Weiyong Shen, Svetlana Cherepanoff, Fanfan Zhou, Shaoxue Zeng, Jianhai Du, Mark C Gillies

**Affiliations:** 1 Save Sight Institute, Sydney Medical School, Faculty of Medicine and HealthThe University of SydneySydneyAustralia; 2 School of Optometry and Vision Sciences, University of New South WalesSydneyAustralia; 3 Clinical Genomics LaboratorySidra MedicineDohaQatar; 4 Department of Anatomical PathologySt Vincent’s HospitalDarlinghurstAustralia; 5 Faculty of PharmacyThe University of SydneySydneyAustralia; 6 Department of OphthalmologyWest Virginia UniversityMorgantownUnited States; 7 Department of BiochemistryWest Virginia UniversityMorgantownUnited States; Vollum InstituteUnited States; Johns Hopkins University School of MedicineUnited States

**Keywords:** de novo serine synthesis, macula, Müller cells, phosphoglycerate dehydrogenase, reactive oxygen species, Other

## Abstract

The human macula is more susceptible than the peripheral retina to developing blinding conditions such as age-related macular degeneration, diabetic retinopathy. A key difference between them may be the nature of their Müller cells. We found primary cultured Müller cells from macula and peripheral retina display significant morphological and transcriptomic differences. Macular Müller cells expressed more phosphoglycerate dehydrogenase (PHGDH, a rate-limiting enzyme in serine synthesis) than peripheral Müller cells. The serine synthesis, glycolytic and mitochondrial function were more activated in macular than peripheral Müller cells. Serine biosynthesis is critical in defending against oxidative stress. Intracellular reactive oxygen species and glutathione levels were increased in primary cultured macular Müller cells which were more susceptible to oxidative stress after inhibition of PHGDH. Our findings indicate serine biosynthesis is a critical part of the macular defence against oxidative stress and suggest dysregulation of this pathway as a potential cause of macular pathology.

## Introduction

The macula, a specialized region located at the posterior pole of the primate retina, has the greatest density of cone photoreceptors for the highest visual acuity (Sharon et al., 2002). The impact of common pathologies such as age-related macular degeneration (AMD), diabetic retinopathy (DR) and macular telangiectasia type 2 (MacTel) are most devastating at the macula, leading to vision loss and blindness. Understanding the unique biochemical and anatomic specializations of the macula may provide new ways to prevent and treat these diseases.

A major difference between the macula and peripheral retina is the density of the retinal neurons. Although the macula occupies less than 1.4% of the retina area, it contains ~8.4% of cones,~3.4% of rods and ~60% of the ganglion cells (Curcio and Allen, 1990). The ratio of cones to rods is much higher in the macula (1:8) than that in the peripheral retina (1:20) (Curcio et al., 1990). The high density of neurons in the macula is associated with a high metabolic rate and increased levels of oxidative stress (Handa, 2012).

Müller cells, the major glial cells of retina, are responsible for retinal redox homeostasis and metabolic support of retinal neurons (Reichenbach and Bringmann, 2013). They control the composition of retinal extracellular fluids by mediating the transcellular transport of ions, water and bicarbonate (Bringmann et al., 2005; Kofuji and Newman, 2004; Newman, 1996). They may play an important role in retinal glucose metabolism and provide substrates to photoreceptors, one of the most metabolically active cell types in the body (Poitry-Yamate et al., 1995). Müller cells also promote photoreceptor survival by providing neurotrophic substances and removing metabolic end products (Poitry et al., 2000). Whether these functional requirements of Müller cells differ in the macula and the retinal periphery is unknown.

Müller cells in the macula display unique morphological features. The apical processes of all Müller cells envelop the photoreceptor cell bodies within the outer nuclear layer as the outer limiting membrane, extending distally to form the ‘fibre baskets’ (Bringmann et al., 2006). Müller cells in the periphery share most of these features. Foveal Müller cells are Z-shaped: their processes run vertically from the inner limiting membrane (ILM) to the inner nuclear layer (INL), then horizontally away from the fovea within Henle’s fibre layer (HFL) then vertically again to the outer limiting membrane (OLM). A specialized, cone-shaped zone of Müller cells described in the central and inner part of the fovea has recently received renewed attention (Gass, 1999; Yamada, 1969).

Macular and peripheral Müller cells may also differentially express several functional proteins. Müller cells from the macula and the periphery both express CD117 but only peripheral Müller cells express CD44 (Too et al., 2017). Macular Müller cells also express more Aquaporin-4 than peripheral Müller cells, which may contribute to a specialized ‘glymphatic system’ of the macula (Daruich et al., 2018).

The importance of Müller cells for retinal homeostasis suggests that their dysfunction contributes to many retinal diseases. Retinal oedema and neuronal degeneration, the common features of many retinal diseases, may occur secondary to abnormal retinal glutamate metabolism and ionic disturbances caused by Müller cell dysfunction (Bringmann et al., 2006). Müller cell dysfunction has been implicated in MacTel) (Powner et al., 2010) and in a form of autoimmune retinopathy (Peek et al., 1998). Any primary or secondary impairment of Müller cells may cause or at least aggravate the degeneration of retinal neurons by increasing their susceptibility to noxious stimuli. Recent report suggests that Müller cells are the major sites in the retina of de novo synthesis of serine and glutathione which support retinal neurons and protect them from oxidative stress (Zhang et al., 2018). Therefore, we investigated molecular and functional differences of Müller cells in the macula and peripheral regions of retina, with particular interest in serine and glycine metabolism.

## Results

### Isolation, culturing and validation of primary Müller cells from human macula and peripheral retina

**Figure 1.**
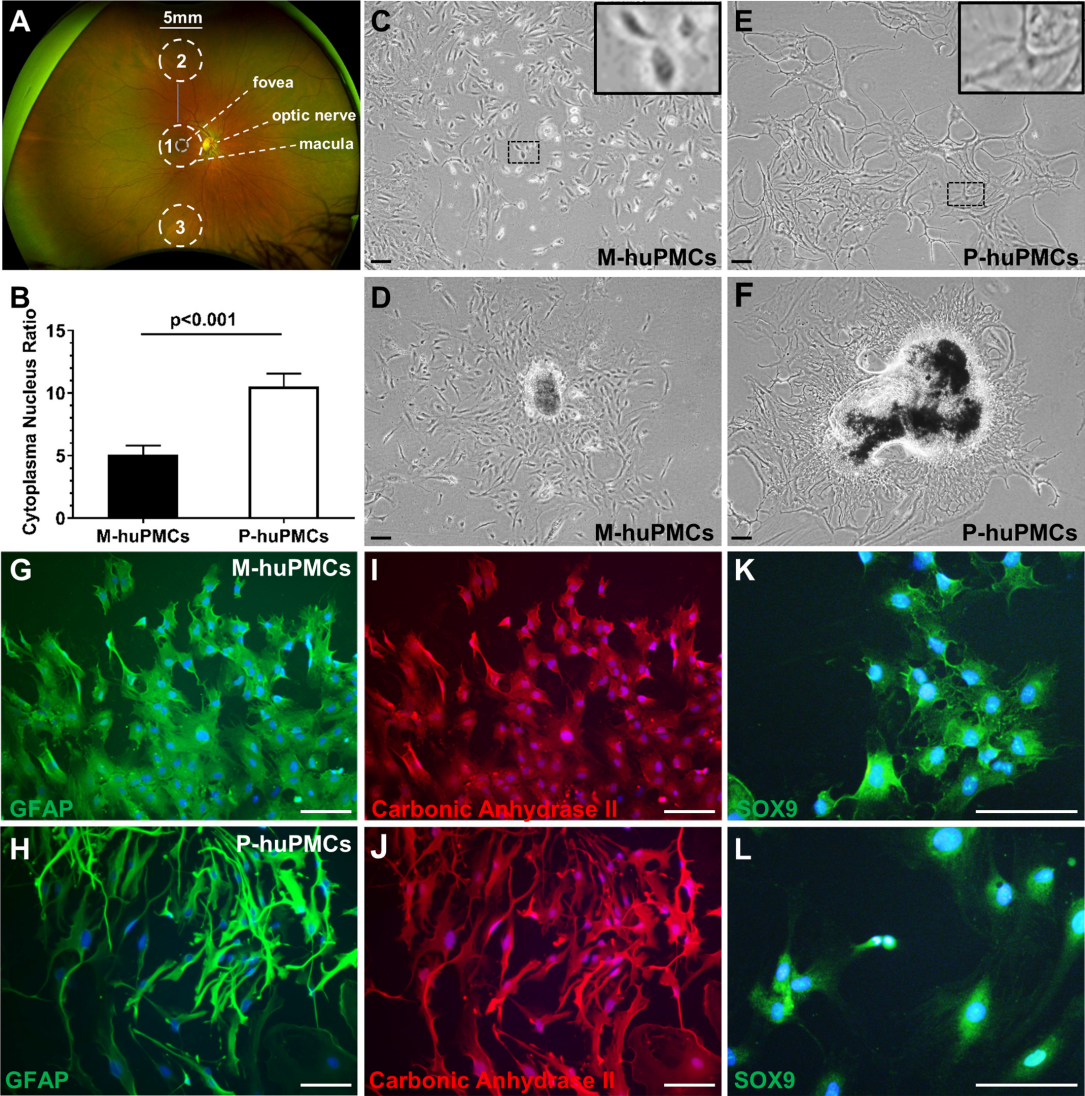
Morphology of primary macular and peripheral Müller cells. (**A**) A wide-field fundus image showing the retinal areas used for primary Müller cell culture. Retinal tissue at Area one was used to derive primary Müller cells from the macula while the punches from Areas 2 and 3 were pooled to derive primary Müller cells from the peripheral retina. (**B**) Cytoplasm/nucleus ratio of primary Müller cells from the macula (M-huPMCs) and peripheral (P-huPMCs) human retina (n = 8). (**C-F**) Bright field images of huPMCs isolated from the macula (**C-D**) or peripheral (**E-F**) retina. (**D**) Primary Müller cells that grew out from macular retinal piece. (**F**) Primary Müller cells that grew out from peripheral retinal piece. (**G-L**) M-huPMCs (upper panel) and P-huPMCs (lower panel) (P0) without sub-culturing expressed three specific protein markers of Müller cells: GFAP (**G-H**), carbonic anhydrase II (**I-J**) and SOX9 (**K-L**). Black scale bar = 200 µM, white scale bar = 100 µM. 10.7554/eLife.43598.004Figure 1—source data 1.Source data for Figure 1B.

We isolated macular and mid-peripheral retinal pieces from healthy human donors’ eyes and cultured the Müller cells as previously described (Zhu et al., 2015) (Figure 1A). The anatomical boundary of the macula is commonly recognized as a region approximately 5.5-mm-diameter, centred at the fovea (Chopdar et al., 2003). We thus trephined 5 mm diameter disks centred at the fovea and similarly sized pieces from mid-peripheral retina (4.5 mm to 7.5 mm from the fovea) (Mori et al., 2016). After 4–6 weeks in culture, Müller cells that grew out from the macular (macular-human primary Müller cells [M-huPMCs]) and peripheral (peripheral-human primary Müller cells [P-huPMCs]) retinal pieces had distinct cell morphologies (Figure 1D & F). M-huPMCs were small, spindle- or stellate-shaped cells with a low cytoplasm/nucleus ratio (Figure 1B & C); the P-huPMCs had large cell bodies, multiple cytoplasmic processes and a higher cytoplasm/nucleus ratio (Figure 1B & E). Both M-huPMCs and P-huPMCs without sub-culturing (P0) demonstrated positive immunolabeling for four Müller-cell-specific markers: Glial fibrillary acidic protein (GFAP) (Figure 1G–H), carbonic anhydrase II (Figure 1I–J), SOX9 (Figure 1K–L) (Song et al., 2013) and Cellular Retinaldehyde Binding Protein (CRALBP) (Figure 1—figure supplement 1). GFAP is a non-specific response of Müller cell stress which they usually express in cell culture (Augustine et al., 2018). This confirmed that these two morphologically distinct cell populations are Müller cells.

**Figure 1—figure supplement 1.**
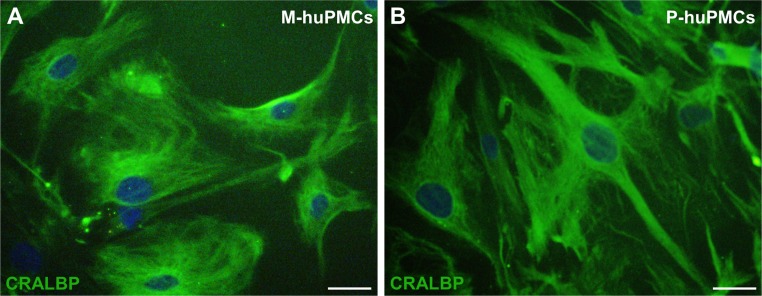
CRALBP immunostaining of M-huPMCs and P-huPMCs. M-huPMCs (**A**) and P-huPMCs (**B**) at P0 passage both expressed CRALBP, the specific protein marker of Müller cells. Scale bar = 50 µM (M = macula; p=peripheral).

### Transcription profiles of macular and peripheral Müller cells

**Figure 2.**
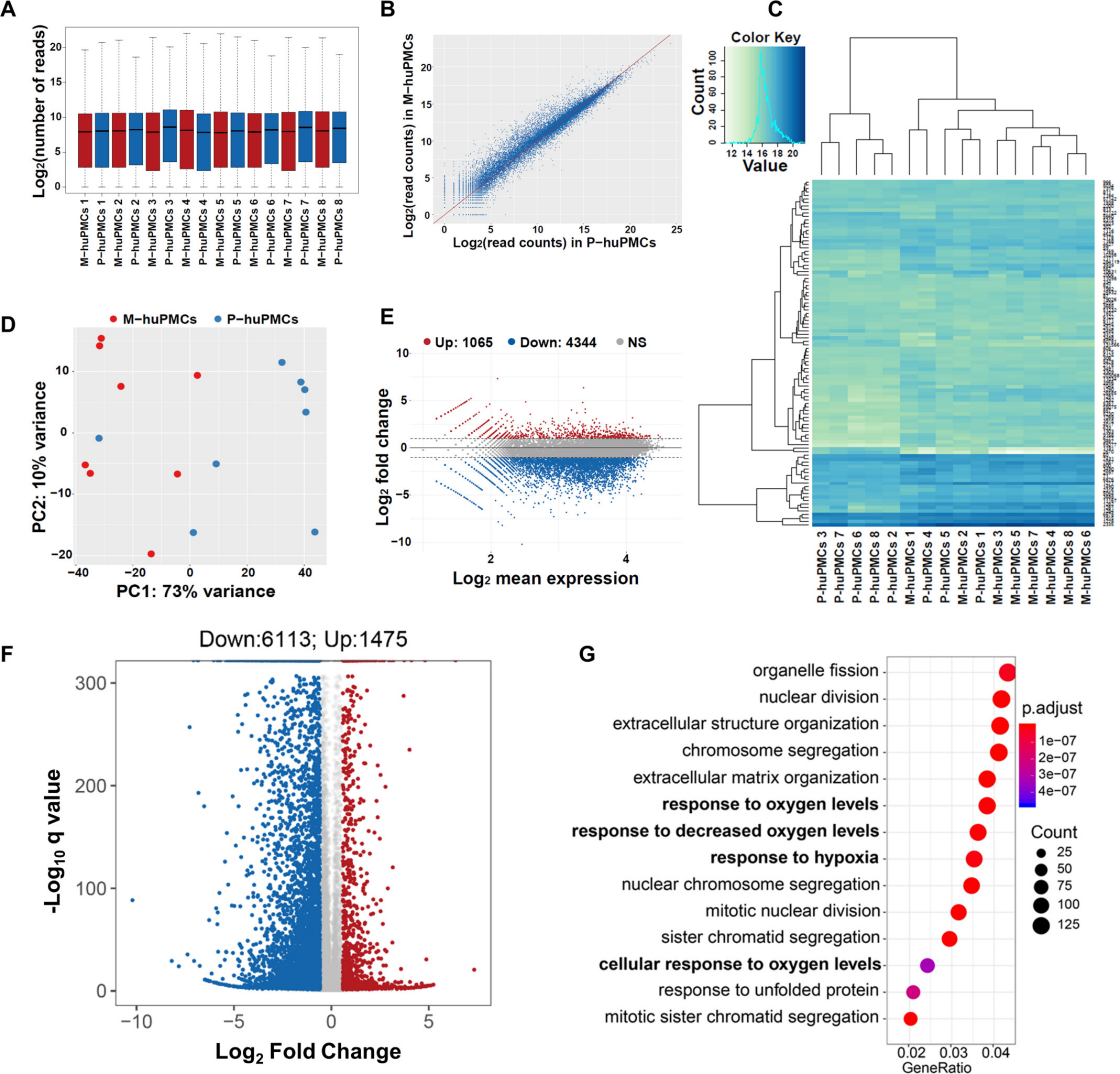
Transcription profiles of macular and peripheral Müller cells. (**A**) Boxplot of the Reads expression values of the M-huPMCs and P-huPMCs cultured from eight donor retinas. (**B**) Scatter Plot for correlations of genes expressed in the M-huPMCs and P-huPMCs. (**C**) Heatmap of differentially expressed genes between the M-huPMCs and P-huPMCs of eight donors shows the top 100 genes with the smallest q-values. (**D**) Principal component analysis (PCA) was performed with the RNA-seq data derived from the M-huPMCs and P-huPMCs. (**E**) Bland–Altman (MA) plot of differentially expressed genes in the M-huPMCs and P-huPMCs. (**F**) Volcano plot of differentially expressed genes in the M-huPMCs and P-huPMCs. The log 10 (q values) were plotted against the log 2 (Fold Change) in gene expression. The genes upregulated (n = 1475) 1.5-fold or more and with an FDR corrected p-value<0.05 were depicted as red dots; while the genes downregulated (n = 6113) by 1.5-folds or more and with a FDR corrected p-value<0.05 were depicted as blue dots. (**G**) Gene ontology (GO) analysis of upregulated genes in M-huPMCs relative to P-huPMCs. The top 14 GO terms in the biological process category are displayed and ordered by enriched gene number and adjusted p value.

RNA-sequencing analysis of matched M-huPMCs (n = 8) vs P-huPMCs (n = 8) from each individual retina of eight donors revealed significant differences between the two cell types. Reads expression values of all 16 samples suggested a highly similar gene expression pattern (Figure 2A). Scatter plots show the correlation of the gene expression profiles between M-huPMCs and P-huPMCs (Figure 2B). In Figure 2C heatmap of gene expressions of M-huPMCs and P-huPMCs (R, DESeq2) is used to visualize the top 100 genes with the smallest q-values. Pairwise sample distances were estimated using the Euclidean distance and sample clustering was done with the Ward algorithm. Principal component analysis (PCA) shows the clustering of M-huPMCs and P-huPMCs (Figure 2D). A total of 7588 differentially expressed genes (DEGs) were identified in Müller cells isolated from human macula and peripheral retina with a 1.5-fold or more increase or decrease and with an FDR corrected p-value<0.05 (Figure 2F, Supplementary **S1** and **S2**). Bland–Altman (MA) plot and Volcano plot show the differentially expressed genes in M-huPMCs and P-huPMCs (Figure 2E–F). Based on gene ontology (GO) enrichment analysis (ClusterProfiler), these upregulated genes were significantly associated with numerous GO Biological process terms, including ‘response to oxygen levels’, ‘response to decreased oxygen levels’ and ‘response to hypoxia’ (Figure 2G).

RNA sequencing found that both the M-huPMCs and P-huPMCs strongly expressed (high RPKM number [Reads Per Kilobase of transcript per Million mapped reads]) gene markers for the Müller cell: vimentin, glutamine synthetase (GS), CRALBP, clusterin, carbonic anhydrase II and GFAP (Table 1) and negligibly expression (low RPKM number) of gene markers of the other retinal cell types: rhodopsin for photoreceptors; mGluR6 for the bipolar cells, syntaxin 1A for the amacrine cells, Iba1 for the microglia cells and PECAM1 for vascular endothelial cells (Table 1). These findings are consistent with Müller cells retaining their original genotype in primary tissue culture. Notably, the expression of Müller cell markers was higher in the P-huPMCs compared to M-huPMCs.

**Table 1.** Gene expression of retinal cells marker in identified cells from macula and peripheral retina

Marker	Gene name	Gene ID	Length	M-rpkm	P-rpkm	Log_2_ratio(P/M)	Regulation(P/M)	p-value
Müller cell	Vimentin	7431	2151	2923.82	3492.22	0.26	up	<2.22E-308
	Glutamine synthetase	2752	4737	23.46	95.31	2.02	up	<2.22E-308
	RLBP1 (CRALBP)	6017	1752	4.50	34.79	2.95	up	<2.22E-308
	CLU clusterin	1191	3012	78.80	763.72	3.28	up	<2.22E-308
	Carbonic anhydrase II	760	1666	15.52	138.45	3.16	up	<2.22E-308
	GFAP	2670	3097	31.87	944.58	4.89	up	<2.22E-308
Other cell types								
Photoreceptors	RHO rhodopsin	6010	2768	0.01	0.05	2.13	up	1.43E-07
Biopolar cells	mGluR6	2916	6143	0.03	0.31	3.2	up	3.13E-134
Amacrine cells	STX1A syntaxin 1A	6804	2138	4.35	3.89	−0.16	down	3.43E-06
Microglia	Iba1	199	1491	1.44	3.67	1.35	up	3.99E-129
Endothelial	PECAM1	5175	6831	0.25	0.72	1.52	up	1.21E-138

### Differential expression of genes related to the de novo serine synthesis pathway in Müller cells isolated from the human macula and peripheral retina

MacTel is a retinal disease which may be caused primarily by dysfunction of macular Müller cells. A recent study reported that the de novo serine metabolic pathway may play an important role in MacTel through defects of phosphoglycerate dehydrogenase (PHGDH), a key rate-limiting enzyme in de novo serine metabolism (Scerri et al., 2017). We have reported that PHGDH is predominantly expressed in human Müller cells and that inhibition of PHGDH aggravates mitochondrial and oxidative stress (Zhang et al., 2018). We found in the present study that the expression of key genes related to de novo serine synthesis (e.g. PHGDH, PSAT1 and SHMT2) was higher in M-huPMCs than in P-huPMCs (Table 2).

**Table 2.** Serine synthesis pathway-related gene expression in Müller cell isolated from macula and peripheral retina.

Gene name	Gene ID	Length	M-rpkm	P-rpkm	Log_2_ratio (M/P)	Regulation (M/P)	p-value
**PHGDH**	26227	2021	60.36	40.09	0.59	up	<2.22E-308
**PSAT1**	29968	2221	101.15	79.10	0.35	up	<2.22E-308
**PSPH**	5723	2142	12.51	14.20	−0.18	down	2.56E-21
**SHMT1**	6470	2540	3.72	7.05	−0.92	down	2.99E-228
**SHMT2**	6472	2357	64.83	61.14	0.08	up	1.27E-23

**Figure 3.**
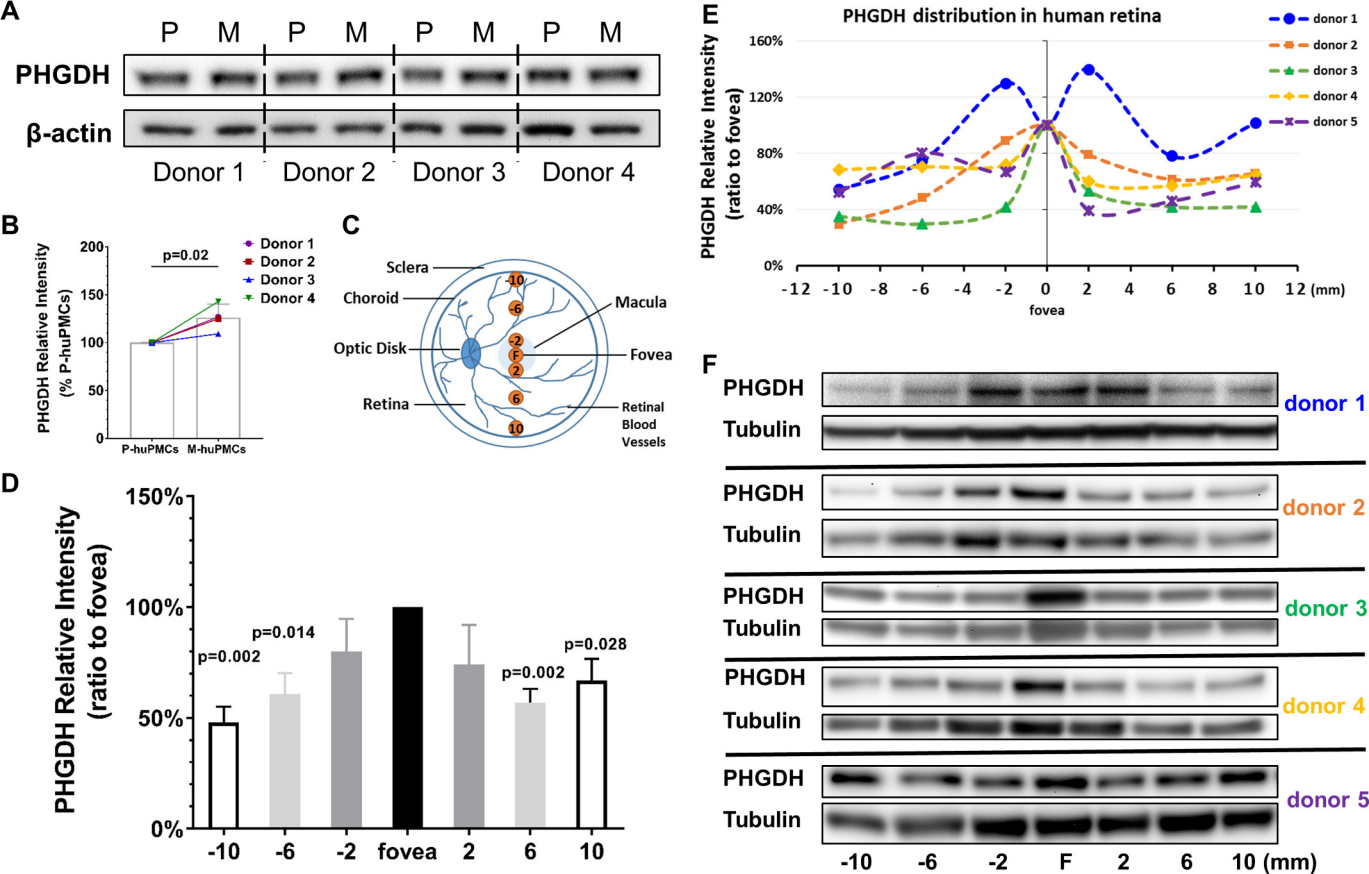
PHGDH expression in cultured Müller cells and retinal tissue from macula and periphery. (**A**) Representative immunoblot showing PHGDH protein expression in primary cultures of human Müller cells from macula and peripheral retina; (**B**) Quantitative analysis of PHGDH relative to β-actin for immunoblots (n = 4 donor retinas); (**C**) Schema of trephined retina area used for Western blotting shown in (**F**); (**D-E**) Quantitative analysis of the expression of PHGDH correlated to different areas of human retina (five donors); (**F**) Expression of PHGDH in different retinal locations shown using western blotting (n = 5 donors). 10.7554/eLife.43598.010Figure 3—source data 1.Source data for Figure 3B,D,E,F.

Western blot studies found that M-huPMCs expressed significantly more PHGDH compared to P-huPMCs (Figure 3A–B, normalized to β-actin, n = 4 donor retinas). We further explored the topographical protein expression of PHGDH using a series of 2 mm fresh retinal tissue punches at different locations across the human retina (Figure 3C). We found that macula expressed significantly more PHGDH (Figure 3D–F, normalized to α/β-Tubulin or Figure 3—figure supplement 1, normalized to total loaded retinal protein) than punches from the peripheral retina, although there were inter-individual variations between the five retinas. We believe that PHGDH expression from the retina largely reflects its expression by Müller cells since they are the main source of PHGDH in the human retina (Zhang et al., 2018).

**Figure 3—figure supplement 1.**
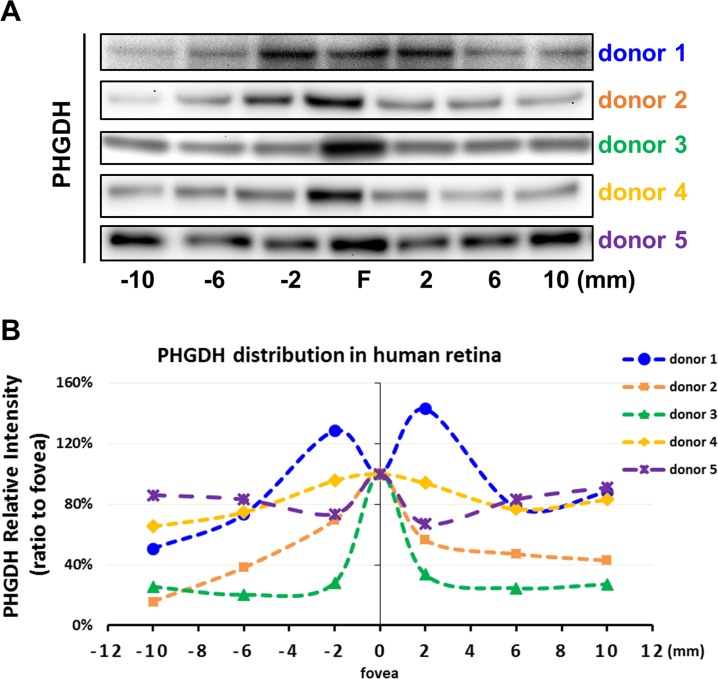
PHGDH expression in the macula and peripheral retina. (**A**) PHGDH expression in different retinal locations was assessed with western blot (n = 5 donors). (**B**) Quantitative analysis of PHGDH expression (normalized to total loaded retinal protein) was correlated with different areas of human retina (n = 5 donors).

**Figure 4.**
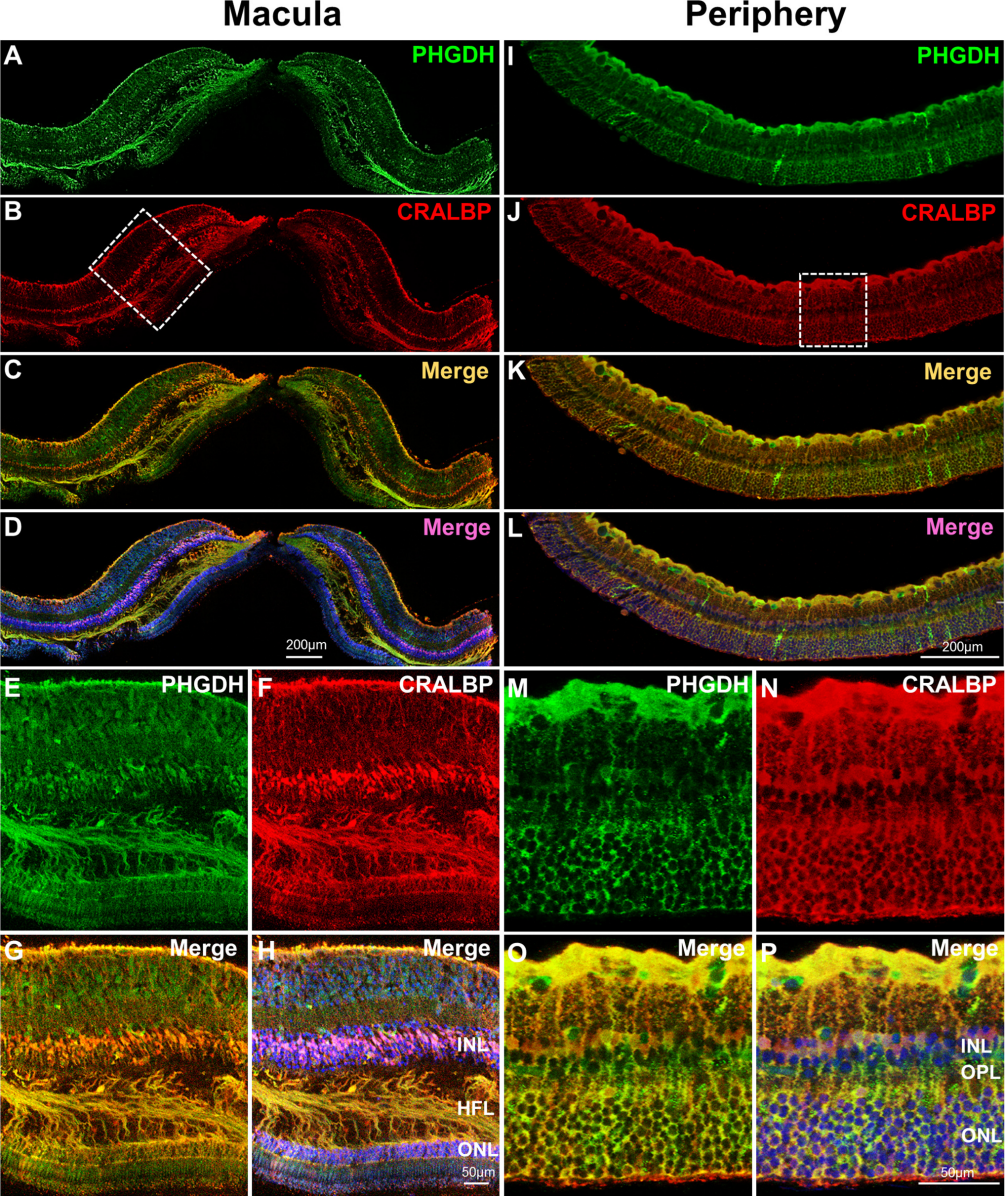
Immunofluorescent staining of PHGDH in human retina. Representative images of immunofluorescence visualization of PHGDH (green) and CRALBP (red, a Müller cell marker) in human macular (**A-H**) and peripheral retina (**I-P**). **E-H** Field enlarged image from **B** (white-dotted box) in macula; **M-P**. Enlarged images from **J** (white-dotted box) in peripheral retina. The specific immunoreactivity of PHGDH antibody was verified in PHGDH knockdown in MIO-M1 cells (Figure 4—figure supplement 1).

Immunofluorescent staining of human macular (Figure 4A–H) and peripheral (Figure 4I–P) retina from the same eye revealed co-localization of CRALBP (red, a Müller cell marker) and PHGDH (green). This is consistent with our previous study that PHGDH in the retina is predominantly expressed in the Müller cells(Zhang et al., 2018). In contrast to the peripheral retina (Figure 4M–P), the macula has multiple layers of Müller cell nuclei in the inner nuclear layer and strongly expresses PHGDH in Henle fibre layer (Figure 4E–H).

**Figure 4—figure supplement 1.**
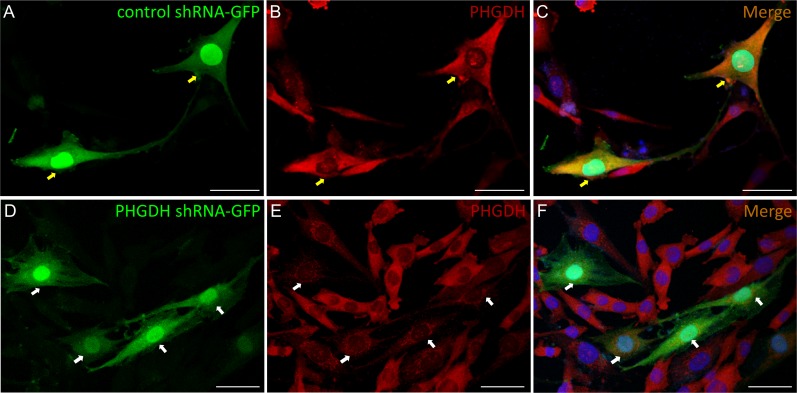
Verification of PHGDH specific immunoreactivity by PHGDH knockdown in MIO-M1 cells. Cells expressing green fluorescence have been successfully transfected with control (**A**) or PHGDH (**D**) shRNA-GFP plasmid. PHGDH was immunostained with anti-PHGDH antibody, visualised with Alexa 594 (red) in the MIO-M1 cells (**B and E**). (**C**) Merged images of **A and B** showing normal PHGDH immunoreactivity in cells transfected with negative control shRNA-GFP plasmid (yellow arrows). (**F**) Merged images of **D and E** showing reduced PHGDH immunoactivity in the cells transfected with PHGDH-shRNA-GFP plasmid (white arrows). Scale bar = 50 µM.

### Metabolic differences between the macular and peripheral Müller cells

**Figure 5.**
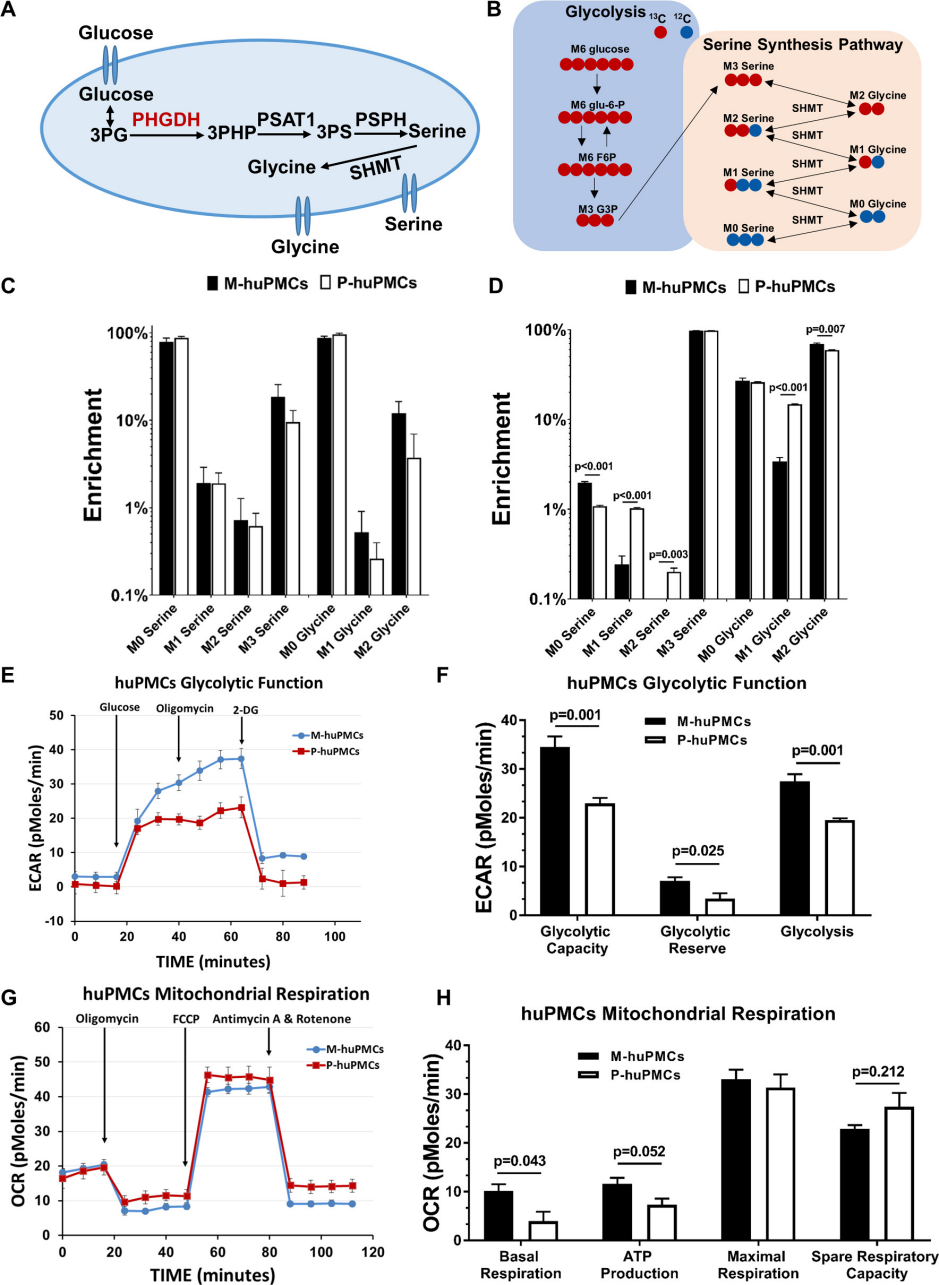
Metabolic differences between the macular and peripheral Müller cells. (**A**) de novo serine/glycine synthesis pathway. 3PG: 3-phosphoglycerate, PHGDH: 3-phosphoglycerate dehydrogenase, PSAT1: phosphoserine aminotransferase 1, PSPH: phosphoserine phosphatase, SHMT: serine hydroxymethyltransferase. (**B**) Simplified schematic of steps in de novo serine/glycine synthesis, showing ^13^C labeling patterns resulting from ^13^C6 glucose substrate. Red fills indicate ^13^C atoms. (**C–D**) ^13^C-serine/glycine levels in M-huPMCs and P-huPMCs after treatment with ^13^C-glucose (**C**) or ^13^C-serine (**D**). (**E–H**) Seahorse XF analysis of M-huPMCs and P-huPMCs. Glycolytic (**E–F**) and mitochondrial functions (**G–H**) were evaluated in human Müller cells in primary culture isolated from macula and peripheral regions (n = 4). 10.7554/eLife.43598.015Figure 5—source data 1.Source data for Figure 5C,D,F,H.

To evaluate the metabolism of differential expression of PHGDH in the macular and peripheral Müller cells, we treated M-huPMCs and P-huPMCs from four different donors with ^13^C-glucose to measure the metabolic flux of serine and glycine from glucose (Figure 5A–B). Following the treatment, different ^13^C labelled serine isotopologue (M0, M1, M2, and M3) and glycine isotopologue (M0, M1 and M2) were quantified with gas chromatography–mass spectrometry (GC-MS). After 3 hr labeling, around 18% of cellular serine (average of both M-huPMCs and P-huPMCs) was replaced by ^13^C-glucose (Figure 5C). M-huPMCs had higher ^13^C labelled serine (24%) and ^13^C-labeled glycine (12%) than of P-huPMCs (13% and 5% respectively, Figure 5C). We did not observe a significant change in the relative level of total serine or glycine in M-huPMCs and P-huPMCs (Figure 5—figure supplement 1).

**Figure 5—figure supplement 1.**
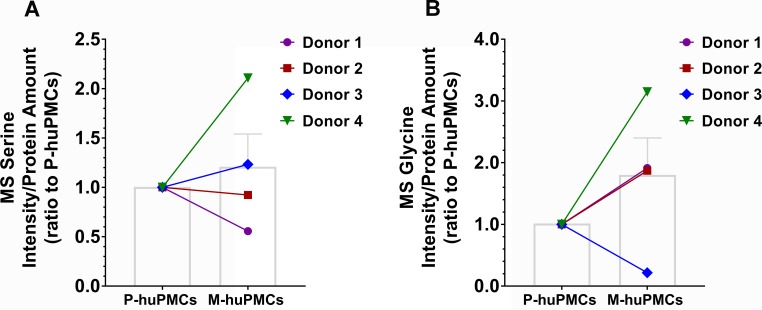
The ratio of serine (**A**) or glycine (**B**) MS intensity to total amount of cellular protein in M-huPMCs and P-huPMCs from the same donor.

We also treated M-huPMCs and P-huPMCs with ^13^C-labeled serine to trace how human primary Müller cells use exogenous serine. After treatment with ^13^C-serine for 24 hr, we found that most ^13^C-serine was converted to ^13^C-glycine but we found no other glucose intermediates, in contrast to previous reports in cancer cells (Amelio et al., 2014). A modest but statistically significantly higher level of ^13^C-labeled M2 glycine (70% vs 59%) suggests that more serine is converted to glycine in M-huPMCs than in P-huPMCs. However, less ^13^C-labeled M1 glycine, M1 serine and M2 serine in M-huPMCs indicates that there is a lower rate of reverse serine/glycine reactions through SHMT occurring in these cells than in P-huPMCs (Figure 5D).

The expression of PHGDH is not only important to the de novo serine synthesis metabolic pathway, but also closely related to cellular glycolytic and mitochondrial functions. We further assessed the glycolytic and mitochondrial functions of M-huPMCs and P-huPMCs using Seahorse Assay. We found that M-huPMCs had significantly higher glycolytic capacity and reserve than P-huPMCs (Figure 5E–F). M-huPMCs also had significantly higher basal mitochondrial respiration and ATP production but lower spare respiratory capacity than P-huPMCs (Figure 5G–H).

### Susceptibility of the M-huPMCs and P-huPMCs to oxidative stress after inhibition of PHGDH

**Figure 6.**
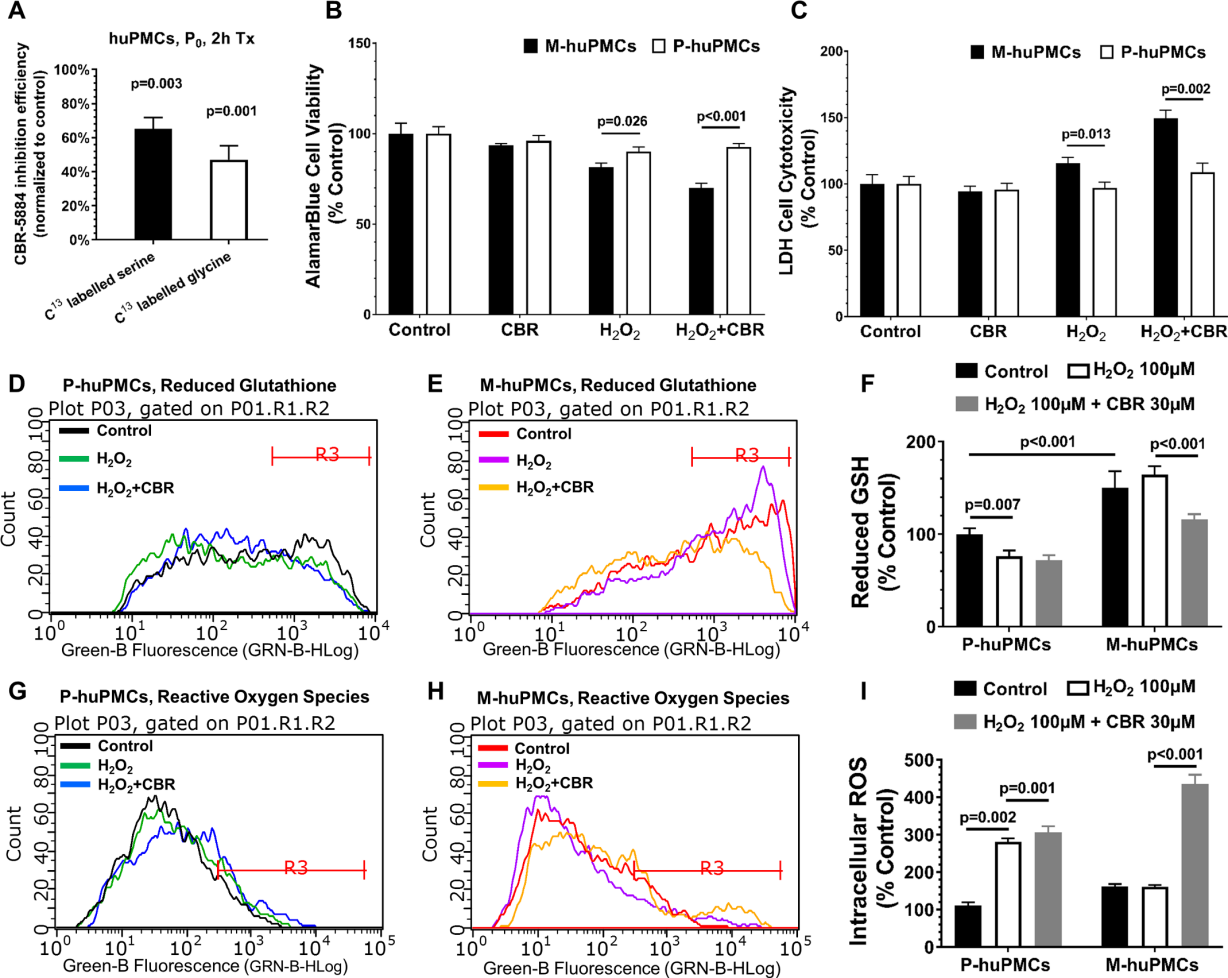
Responses to oxidative stress of primary cultured Müller cells from macula and peripheral retina. (**A**) Measurement of ^13^C-serine and -glycine after PHGDH inhibition in human primary Müller cells; **B-C**. Cell metabolic activity (**B**) and cellular damage (**C**) in the M-huPMCs and P-huPMCs with or without PHGDH inhibition under oxidative stress; **D-I**. Flow cytometry analysis for M-huPMCs and P-huPMCs stained with Thiol Green Dye to detect GSH (**D-F**) and with CM-H_2_DCFDA to evaluate ROS (**G-I**) for cells under mild oxidative stress (100 μM H_2_O_2_), with or without PHGDH inhibition (n = 5). 10.7554/eLife.43598.018Figure 6—source data 1.Source data for Figure 6A–C,F,I.

To further explore the role of PHGDH in Müller cells from different regions, we used a PHGDH specific inhibitor, CBR-5884, to assess the different responses of Müller cells from different retinal regions. We first validated the inhibition efficiency of CBR-5884 in human primary Müller cells and found that treatment of CBR-5884 in huPMCs for 2 hr resulted in 61% and 42% reductions in serine and glycine levels, respectively (Figure 6A). We also found that M-huPMCs and P-huPMCs had similar levels of cell viability and cytotoxicity after exposure to CBR-5884 (30 µM) for 6 hr. M-huPMCs had significantly lower cell viability and higher cytotoxicity levels than P-huPMCs when they were exposed to exogenous oxidative stress (100 µM H_2_O_2_) (Figure 6B–C). Inhibiting PHGDH using CBR-5884 when cells were exposed to oxidative stress led to more pronounced Müller cells cytotoxicity in the macula compared to the periphery.

**Figure 6—figure supplement 1.**
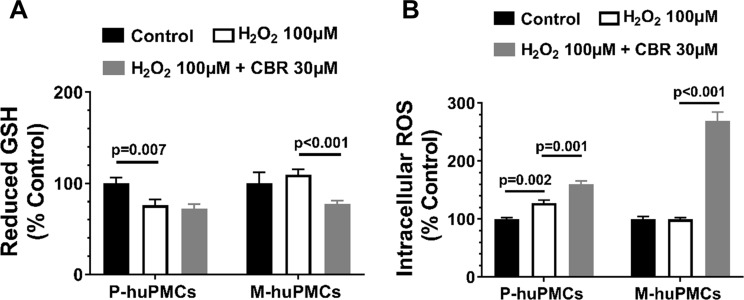
Quantitative re-analysis of the flow cytometry data in Figure 6D–E,G–H. (**A-B**)ROS (CM-H_2_DCFDA) and GSH (Thiol Green Dye) levels in M-huPMCs and P-huPMCs in response to induced hyperoxia with and without CBR, an inhibitor of PHDGH, relative to untreated controls (n = 5 per group).

Since de novo serine synthesis is closely related to the production of glutathione (GSH, the major antioxidant in retina), we speculated that the greater susceptibility of Müller cells to oxidative stress when PHGDH was inhibited, was due to disturbance of the balance between GSH and ROS. We measured GSH and ROS levels in M-huPMCs and P-huPMCs under oxidative stress with or without PHGDH inhibition using flow cytometry (Figure 6D–I). We found significantly higher intracellular levels of GSH in M-huPMCs than in P-huPMCs (Figure 6F). When the cells were also subjected to mild oxidative stress (100 μM H_2_O_2_), P-huPMCs had a more pronounced decrease of GSH levels and elevated ROS levels than M-huPMCs. Inhibition of PHGDH using CBR-5884 induced a more significant GSH reduction in M-huPMCs than in P-huPMCs; ROS levels significantly increased in both M-huPMCs and P-huPMCs (Figure 6F–I, normalized to control of P-huPMCs or Figure 6—figure supplement 1, normalized to P-huPMCs/M-huPMCs own control).

## Discussion

To understand macula pathology, we studied the differences between Müller cells from the peripheral and central retina in primary culture. We found that Müller cells from the macula showed a morphologically distinct response to primary tissue culture compared to those from the peripheral retina. Previous reports have highlighted the morphological differences between Müller cells in the human macula and the retinal periphery in vivo (Bringmann et al., 2018). In situ Müller cells in the foveal region display a Z shape; their elongated processes run with the cone axons in Henle’s fibre layer, most conspicuously in the parafovea (Lujan et al., 2011). In culture, we found that Müller cells isolated from macula were small spindle to stellate shaped cells with lower cytoplasm/nucleus ratios and shorter processes, while the Müller cells from peripheral retina had larger cell bodies, multiple cytoplasmic processes, higher cytoplasm/nucleus ratios. This might be due to the different responses of Müller cells to in vitro culture conditions. These phenotypic differences were retained in the two sub-populations of Müller cells up to the second passage (P2), after which cells showed evidence of division more towards immortalized ‘MIO-M1’-shaped cells. This suggested that these two populations of cells can maintain distinct characteristics up to the second passage, after which they revert to a common phenotype. Therefore, all our following experiments were strictly controlled to use the primary cells up to two passages. Loss of surrounding cells and other in vivo conditions is an acknowledged limitation of tissue culture; however, the different responses of macular *vs*. peripheral Müller cells to the same in vitro conditions suggests a fundamental difference in mechanisms of survival and homeostasis.

While the two types of cells had distinct morphological appearances, immunofluorescent studies confirmed that Müller cells from the macula and retinal periphery both expressed the same range of Müller cell markers (Figure 1); thus, differences in the cytoskeletal and other proteins underpinning these distinct morphologies are yet to be characterized. Our RNA-seq analysis significantly revealed differential gene expression patterns in Müller cells from the macula and peripheral retina. However, both populations of cells strongly expressed Müller cell markers and only weakly expressed markers of other retinal cell types. Interestingly, peripheral Müller cells expressed Müller cell markers more strongly than those from the macula, perhaps in keeping with their ability to maintain a more ‘Müller-like’ morphology in primary culture. Müller cells from the peripheral retina of humans may be more like Müller cells in other species, most of which do not have a macula. It has been reported that human Müller cells from the macula expressed less GS than those from the retinal periphery (Daruich et al., 2018).

We have previously reported that de novo serine synthesis plays important roles in Müller cellular redox homeostasis and mitochondrial function (Zhang et al., 2018). RNA-seq analysis in the present study found that macular Müller cells expressed higher levels of several key enzymes (with high RPKM numbers) involved in this metabolic pathway, such as PHGDH, than peripheral Müller cells. Our Western blot analysis confirmed that the macula expressed more PHGDH than the peripheral retina (Figure 3). The co-localization of PHGDH with the Müller cells marker CRALBP (Figure 4) is also consistent with a previous report that PHGDH is predominantly expressed by Müller cells in retina (Zhang et al., 2018). The strong expression of PHGDH in Henle’s fibre layer, a specialised region of the macula, may explain why macular Müller cells express more PHGDH than those from the retinal periphery.

The macula has the highest metabolic activity in the retina related to the very high density of neurons, especially cone photoreceptors within the fovea (Okawa et al., 2008; Perkins et al., 2004; Perkins and Frey, 2000). As the major supporting glial cell in the retina, it is not surprising that human macular Müller cells display a more active glycolytic and mitochondrial metabolism than peripheral Müller cells. Our Seahorse XF experiments found that macular Müller cells had a significantly higher capacity for basal mitochondrial respiration, and ATP production, as well as higher glycolytic capacity and reserve than peripheral Müller cells (Figure 5). In both experiments, the readings (ECAR or OCR) started at a similar level, indicating that both populations of cells in primary culture had compatible baseline metabolism rates.

The higher expression of PHGDH in macular Müller cells is consistent with more active de novo serine synthesis in the macula than in the retinal periphery. Studies using C^13^-glucose tracer revealed that Müller cells in primary cell culture had very high de novo serine/glycine enrichment (around 18% within 3 hr), much higher than those reported for melanoma cells, where only around 12% of serine was labeled with C^13^-glucose within 24 hr (Scott et al., 2011). We also found that M-huPMCs shifted glucose carbons more toward de novo serine synthesis but less to glycolysis and the TCA cycle than P-huPMCs. Increased activity of the serine pathway in macular Müller cells is consistent with the generally higher metabolic activity in the macula.

We further explored whether the topographic variation in PHGDH expression will affect the responses of Müller cells to exogenous stimuli. Treatment with the PHGDH-specific inhibitor CBR-5884 led to more pronounced cytotoxicity in macular Müller cells than in peripheral Müller cells, after exposure to oxidative stress. This suggests that defects in de novo serine synthesis increase the vulnerability of M-huPMCs to oxidative stress relative to P-huPMCs. This observation aligns with a recent report that PHGDH dysfunction may be related to the pathogenesis of MacTel, a retinal disease characterised by pronounced degeneration of Müller cells that is confined to the macula (Scerri et al., 2017). This study also found serum levels of serine in MacTel patients to be significantly decreased (Scerri et al., 2017). Increased levels of oxidative stress, combined with PHGDH dysfunction, could thus increase the susceptibility of the macula to damage.

The greater rate of de novo serine synthesis in M-huPMCs may be linked to their increased levels of reduced GSH and ROS; this is consistent with higher metabolic activity and oxidative stress in the macula. Inhibition of de novo serine synthesis resulted in a more significant decrease in reduced GSH levels and increase in ROS levels in M-huPMCs than in P-huPMCs. This further supports the hypothesis that impaired de novo serine synthesis can affect ROS balance in macular Müller cells more than in the peripheral retina.

These findings reveal new lines of investigation for understanding the pathogenesis of AMD and DR; the role of Müller cells in both these disease entities has received relatively little attention. The implications for MacTel, potentially a prototypic Müller cell disease (Charbel Issa et al., 2013), are arguably easier to draw. Although MacTel has been reported to be present in 0.1% of the population over 40 years of age, the actual prevalence may be higher (Coorey et al., 2012; Klein et al., 2010). MacTel affects both eyes with a predilection for the temporal parafovea (Gass and Blodi, 1993). Recent clinicopathological studies have consistently found loss of Müller cells markers from the macula. The area of macular luteal pigment depletion, which is a specific and sensitive marker of MacTel clinically, was found to match the area of Müller cell loss, which was in turn associated with photoreceptor degeneration (Powner et al., 2010; Powner et al., 2013). It is now suspected MacTel is caused by a derangement of glial-neural function with a secondary vasculopathy that is not the prime cause of loss of vision. Defects of genes associated with the PHGDH gene have recently been implicated in patients with MacTel (Scerri et al., 2017). Our results indicate that impairment of PHGDH would have more significant effects on macular Müller cells, especially under oxidative stress. Interestingly, we found PHGDH is highly expressed in macular Henle fibre layer, which may explain why MacTel is tightly associated with HFL pathology (Powner et al., 2010).

We have explored the differences between Müller cells from the macula and peripheral human retina. The distinct transcriptional and functional differences we found between these two populations of primary Müller cells contributes to explaining the unique biochemical and metabolic specializations of the human macula. We accept Müller cells in vitro, are different to Müller cells in vivo, since they have lost contact with neurons and blood vessels. Nevertheless, the Müller cells that had been isolated from the different retinal regions responded consistently and differently to the conditions they were exposed to, indicating some molecular differences between the Müller cells from these two different locations. The macula is devastatingly affected by several common blinding conditions including AMD and DR, major causes of vision impairment and loss worldwide. The differences in serine biosynthesis between Müller cells from two regions is consistent with the hypothesis that disease of Macular Müller cells causes metabolic dysfunction which is the primary cause of MacTel. A better understanding of the unique biology of the human macula, both in terms of its resiliency and vulnerability, will significantly inform future approaches to disease prevention and treatments.

## Materials and methods

**Key resources table:** 

Reagent type (species) or resource	Designation	Source or reference	Identifiers	Additional information
Antibody	Rabbit polyclonal anti-3-PGDH	Millipore	Cat# ABS571, RRID:AB_2783876	IF(1:1000), WB (1:1000)
Antibody	Rabbit polyclonalanti-α/β tubulin	Cell Signaling Technology	Cat# 2148, RRID:AB_2288042	WB (1:1000)
Antibody	Mouse monoclonal anti-CRALBP	Abcam	Cat# ab15051, RRID:AB_2269474	IF(1:500)
Antibody	Goat polyclonalanti-GFAP	Abcam	Cat# ab53554, RRID:AB_880202	IF(1:1000)
Antibody	Rabbit polyclonal anti-carbonicanhydrase II	Abcam	Cat# ab6621, RRID:AB_305602	IF(1:1000)
Antibody	Rabbit polyclonal anti-Sox9	Millipore	Cat# AB5535, RRID:AB_2239761	IF(1:1000)
Recombinant DNA reagent	PHGDH-shRNA-GFP plasmid	Sigma Aldrich	MISSION SHRNA PLASMID DNA NM_006623/TRCN0000233032 / -hPGK-Neo - CMV-tGFP	
Recombinant DNA reagent	Negative control-shRNA-GFP plasmid	Sigma Aldrich	MISSION SHRNA CUSTOM DNA NEGATIVE CONTROL - nontarget shRNA (SHC016) - pLKO.1-Neo-CMV-tGFP vector	
Commercial assay or kit	Intracellular GSH detection kit	Abcam	ab112132	
Commercial assay or kit	CM-H_2_DCFDA	Molecular Probes	C6827	
Commercialassay or kit	Pierce LDH cytotoxicity assay kit	ThermoFisher Scientific	88953	
Commercialassay or kit	AamarBlue cellviability reagent	ThermoFisher Scientific	DAL1100	
Chemicalcompound, drug	CBR-5884	Sigma Aldrich	SML1656	
Software,algorithm	SPSS	SPSS	RRID:SCR_002865	

### Isolation, culture and identification of primary Müller cells from the macula and peripheral human retina

Human retinas were obtained from *post-mortem* donor eyes with ethical approval from Human Research Ethics Committee of the University of Sydney (HREC#:16/282). Human retinas without known retinal diseases were isolated as described previously (Zhang et al., 2018). The dissected retina was immersed in DMEM medium in a 92 mm culture dish with transparent background. The *macula lutea* was readily visualized with bright yellow macula pigment. As demonstrated in Figure 1, a 5 mm tissue punch centred on the central retina as well as the superior and inferior mid-periphery was taken. The mid-periphery was defined as the mid-point between the edge of the *macula lutea* and the *ora serrata*. Primary Müller cells were cultured according to our established laboratory protocol (available upon request). After retinal pieces from macula and peripheral region were cultured in DMEM medium for 6–8 weeks (with twice weekly medium change), immunofluorescent staining of Müller cell markers (GFAP, carbonic anhydrase II, SOX9 and CRALBP) was performed on the matched sets of primary Müller cells (P0, without subculturing) isolated from the macula and peripheral regions of each donor eye. Images were taken with the Olympus microscope (IX71).

### RNA sequencing

After extracting the total RNA from M-huPMCs and P-huPMCs (n = 8), mRNA was enriched using the oligo (dT) magnetic beads. The library preparation, sequencing and quality control were commercially contracted to BGI (https://www.bgi.com/global/). The mRNA was fragmented into short fragments (200 ~ 700 bp) in the fragmentation buffer. The first-strand cDNA was synthesized with random hexamer-primer using the mRNA fragments as templates, followed by the second strand synthesis. The double stranded cDNA was purified with a QiaQuick PCR extraction kit and then used for end repair and base A addition. Finally, sequencing adapters were ligated to the fragments. The fragments were purified by SPRI bead size selection and enriched by PCR amplification. The library products were sequenced using Illumina HiSeq 2500 with paired end 100 bp read length.

### RNA data analysis

Primary sequencing data was generated by Illumina HiSeq 2500. Raw reads were filtered to remove adaptor sequences and PCR duplicates. The filtered clean reads were aligned to the reference sequences with SOAP2. The alignment data was utilized to calculate distribution of reads on reference genes and perform coverage analysis. Downstream analysis was performed including gene differential expression analysis (DESeq v1.18.0), alternative splicing (tophat v2.0.8+cufflinks v2.0.2) and SNP detection (using SOAPsnp v1.05). Results of gene expression included gene expression levels and differential expression analysis was performed using DESeq2 in R (version 3.5.1). P-values were adjusted for multiple testing using the Benjamini-Hochberg procedure (Benjamini and Hochberg, 1995). A false discovery rate (FDR) adjusted p-value (i.e. q-value)<0.05 was set for the selection of differential expression genes. Two dimensional plots of principal components were calculated with principal component analysis using R software. We used ClusterProfiler (Bioconductor; https://bioconductor.org/pack-ages/release/bioc/html/clusterProfiler.html) which is an R package to analyse gene clusters and classify biological terms.

### Seahorse XF analysis

Macular and peripheral Müller cells were seeded at a density of 2 × 10^4^ cells/well in DMEM medium into the Seahorse XF96 cell culture microplates (Seahorse Bioscience, Agilent Technologies, Santa Clara, CA, USA). The mitochondrial stress assay was carried out in assay medium containing XF base medium (Aligent). Assay medium was freshly prepared and adjusted to pH 7.4. After 24 hr incubation at 37°C in 5% CO_2_, the confluent cells were ready to be tested. After programming in accordance with manufacturer’s recommendation, the testing plate was placed in Seahorse XF Analyser. Oxygen consumption rates (OCR) and extracellular acidification rates (ECAR) were measured approximately every 8 min. The stress reaction system in each well contained 175 µl assay buffer, 25 µl Oligomycin, 25 µl FCCP and 25 µl Rotenone/Antimycin A to achieve the working concentrations of 1.26 µM, 1 µM, 0.5 µM respectively, and the drugs were sequentially added to each well at different time points according to manufacturer instructions. The OCR and ECAR curves were obtained as the reads of stress resulted from drugs at different time points. The rate is normalized to the amount of protein in each well. The differences in glycolytic and mitochondrial functions were compared between the two primary Müller cell populations (macula and peripheral retina).

### LDH cell cytotoxicity assay and AlamarBlue cell viability assay

M-huPMCs and P-huPMCs (P0) were detached from culture dishes using TrypLE enzyme (Thermo Fisher Scientific) and seeded at a density of 1 × 10^4^ cells/well in 96-well plates. Cells were cultured to 80% confluence in DMEM/10% FBS and then starved overnight with Basal Medium Eagle (Gibco) supplemented with 1% FBS, 1% penicillin-streptomycin (P/S) and 1% Insulin-Transferrin-Selenium (ITS, Thermo Fisher Scientific). M-huPMCs and P-huPMCs (P1) were then treated with 100 μM H_2_O_2_, 15 μM CBR-5884 and 100 μM H_2_O_2_ + 15 μM CBR-5884 for 6 hr, respectively. A medium only, no treatment group was included as a control. The LDH concentration in each group was assayed by LDH cytotoxicity assay kit (Pierce). In brief, 20 µl supernatant of the 96-well plate was transferred into a 386-well plate. 20 µl reaction mixture was added into each well of the plate. The mixture was incubated at room temperature for 30 min. 20 µl stop solution was added to cease the reaction. The absorbance was measured at 490 nm and 680 nm with the plate reader (Safire^2^, Tecan). To assess cell viability, 15 µl alamarBlue reagent was added to 150 µl medium in each well of the 96-well plate. After a 2 hr incubation at 37°C, the absorbance was read at 570 and 600 nm with the Tecan Safire plate reader (TECAN).

### Western blot

The cells were washed with PBS and lysed in RIPA buffer (Sigma Aldrich) with Protease/Phosphatase Inhibitor Cocktail (Cell Signalling). The lysed cells were incubated at 4°C for 10 min and then centrifuged at 12,000 g at 4°C for 10 min. The supernatants were collected and protein concentrations were determined using a BCA protein assay kit (Thermo Fisher Scientific). Samples were heated with reducing buffer and NuPAGE 4–12% Bis-Tris Protein Gels (Life Technologies) at 70°C for 10 min. The samples were loaded onto NuPAGE 4–12% Bis-Tris Protein gels (Thermo Fisher Scientific) and electrophoresed at 180V, 4°C for 70 min. The proteins were transferred using the iBlot gel transfer device (Thermo Fisher Scientific). The PVDF membranes (Millipore, 0.42 uM) were blocked with 5% bovine serum albumin (BSA), Tris-Buffered Saline and 0.1% Tween 20 (TBST) for 1 hr at room temperature (RT) and then incubated with either primary antibody overnight at 4°C: PHGDH (Millipore, #ABS571, 1:1000) or α/β tubulin (Cell Signalling, #2148, 1:1000). The membranes were washed three times with PBST and incubated with the HRP-conjugated secondary antibody (1:5000) at RT for 2 hr on the following day. Then the membranes were washed with TBST for 5 min, three times and TBS for 5 min once. After washing, the membranes were incubated in Clarity Western ECL Substrate for 5 min and imaged using the G-Box imaging system (In Vitro Technologies).

### Vibratome sectioning

Retinal punches from the macula and peripheral regions of donor eyes were fixed in 4% paraformaldehyde (PFA) in PBS for 2 hr and then transferred to PBS for 1 hr. Retinal pieces were embedded in low melting point agarose (42°C, 3% agarose (Lonza) in PBS). Tissue blocks were cooled down at 4°C until the agarose solidified. Agarose blocks were trimmed and glued to the metal chuck of a vibratome (Leica, VT1200S). Vibratome sections (100 μm thick) were obtained from retinal tissues and stored in PBS at 4°C.

### Immunofluorescent staining

HuPMCs in 24-well plates were fixed in 4% PFA for 1 hr, then rinsed and stored in PBS for later immunofluorescence staining. Retinal vibratome sections and huPMCs were initially blocked in 5% normal goat serum (Sigma) overnight at 4°C. Sections were immunostained with primary antibodies: PHGDH (Millipore, ABS571, 1:1000), CRALBP (Abcam, ab15051, 1:500), GFAP (Abcam, ab53554, 1:1000), Carbonic Anhydrase II (Abcam, ab6621, 1:1000), SOX9 (Millipore, ab5535, 1:1000) diluted in PBS containing 1% normal goat serum and 0.5% Triton X-100. Vibratome sections were incubated for 4 nights at 4°C. Thereafter, huPMCs were incubated with species-specific secondary antibodies conjugated with Alexa Fluor 488 (green) or 594 (red) (Molecular Probes) at a 1:1000 dilution for 2 hr at room temperature; while the vibratome sections were incubated at 4°C overnight. Cells and sections were then rinsed with PBS and nuclei were stained with Hoechst 33342 (Thermo Fisher Scientific). After mounting in VECTASHIELD antifade mounting medium (Vector Laboratories), immunofluorescence labelling of huPMCs and vibratome sections was captured with the ZEISS confocal laser-scanning microscope (LSM700, Carl Zeiss) and ZEN Blue software.

### ^13^C Glucose labeling experiments

M-huPMCs and P-huPMCs (P0) in T25 flasks (Corning) were starved overnight with BME supplemented with 1%FBS, 1% penicillin-streptomycin, and 1% Insulin-Transferrin-Selenium (ITS). M-huPMCs and P-huPMCs were treated with 30 µM CBR-5884 for 4 hr. Cells were incubated with ^13^C-glucose (5 mM, CLM-1396–0.5, Cambridge Isotope Lab) with or without CBR-5884 (30 µM) in KRB (Krebs-Ringer-Bicarbonate buffer) for 2 hr in a CO_2_ incubator. Before the incubation, KRB containing the ^13^C-glucoce was pre-warmed at 37°C in a CO_2_ incubator for 2 hr. 1 ml cold 0.9% sodium chloride was used to rinse the cells, followed by 800 µl extraction buffer (methanol: ddH_2_O, 800:200). Cells were then scraped off the flask on dry ice and transferred into Eppendorf tubes. The cell/extraction buffer mix was homogenized and placed on dry ice for 30 min, then centrifuged at 13,000 g for 10 min at 4°C, and the supernatant transferred into the clean tubes. 0.1 M NaOH was added into the pellet of and incubate at 37°C for 16 hr. Protein concentration was measured by BCA method (Thermo Fisher Scientific) (Du et al., 2015; Zhu et al., 2018).

### Flow cytometry

We detected the intracellular ROS and GSH in M-huPMCs and P-huPMCs (P0) after different treatments, using CM-H_2_DCFDA (Molecular Probes) and Intracellular GSH Detection Kit (Abcam). The huPMCs were washed with PBS to terminate the treatment and TrypLE enzyme (Thermo Fisher Scientific) was used to detach cells from culture dishes. After centrifugation (340 g), cells from different treatments were separated into two groups and incubated with either 2 µM CM-H_2_DCFDA or 1 × Thiol Green Dye for 1 hr in HBSS at 5% CO2, 37°C, respectively. Cells were centrifuged at 340 g for 5 min, washed with PBS and loaded onto a Guava easycyte flow cytometer (Merck Millipore). Cells were gated for singlets (GRN-B-Alog vs. GRN-B-Hlog). The fluorescent event counts were recorded, and the data was analysed using Guava easycyte software 3.1.

### Statistical analysis

The data are expressed as the means ± SEM. Statistical analyses were performed using the SPSS version 17.0 for Windows software. The differences between mean values were evaluated using one-way ANOVA, followed by Tukey post hoc test. Student’s t tests were used to compare differences between any given two groups throughout the study. A p-value less than 0.05 was considered to indicate a statistically significant difference.

## Data Availability

RNA sequencing data are included in the manuscript and Supplementary files. These data are also available at Dryad (https://doi.org/10.5061/dryad.hp60p89). The following dataset was generated: ZhangTZhuLMadiganMLiuWShenWCherepanoffSZhouFDuJGilliesM2018Data from: Human macular Müller cells rely more on serine biosynthesis to combat oxidative stress than those from the peripheryDryad Digital Repository10.5061/dryad.hp60p89PMC653308231036157

## Additional files

Supplementary file 1. RNA sequencing raw data.

Supplementary file 2. RNA sequencing filtered data.

## References

[bib1] Amelio I, Cutruzzolá F, Antonov A, Agostini M, Melino G (2014). Serine and glycine metabolism in cancer. Trends in Biochemical Sciences.

[bib2] Augustine J, Pavlou S, O'Hare M, Harkin K, Stitt A, Curtis T, Xu H, Chen M (2018). Characterization of a spontaneously immortalized murine müller glial cell line QMMuC-1. Investigative Opthalmology & Visual Science.

[bib3] Benjamini Y, Hochberg Y (1995). Controlling the false discovery rate: a practical and powerful approach to multiple testing. Journal of the Royal Statistical Society: Series B.

[bib4] Bringmann A, Uckermann O, Pannicke T, Iandiev I, Reichenbach A, Wiedemann P (2005). Neuronal versus glial cell swelling in the ischaemic retina. Acta Ophthalmologica Scandinavica.

[bib5] Bringmann A, Pannicke T, Grosche J, Francke M, Wiedemann P, Skatchkov SN, Osborne NN, Reichenbach A (2006). Müller cells in the healthy and diseased retina. Progress in Retinal and Eye Research.

[bib6] Bringmann A, Syrbe S, Görner K, Kacza J, Francke M, Wiedemann P, Reichenbach A (2018). The primate fovea: structure, function and development. Progress in Retinal and Eye Research.

[bib7] Charbel Issa P, Gillies MC, Chew EY, Bird AC, Heeren TF, Peto T, Holz FG, Scholl HP (2013). Macular telangiectasia type 2. Progress in Retinal and Eye Research.

[bib8] Chopdar A, Chakravarthy U, Verma D (2003). Age related macular degeneration. Bmj.

[bib9] Coorey NJ, Shen W, Chung SH, Zhu L, Gillies MC (2012). The role of glia in retinal vascular disease. Clinical and Experimental Optometry.

[bib10] Curcio CA, Sloan KR, Kalina RE, Hendrickson AE (1990). Human photoreceptor topography. The Journal of Comparative Neurology.

[bib11] Curcio CA, Allen KA (1990). Topography of ganglion cells in human retina. The Journal of Comparative Neurology.

[bib12] Daruich A, Matet A, Moulin A, Kowalczuk L, Nicolas M, Sellam A, Rothschild PR, Omri S, Gélizé E, Jonet L, Delaunay K, De Kozak Y, Berdugo M, Zhao M, Crisanti P, Behar-Cohen F (2018). Mechanisms of macular edema: beyond the surface. Progress in Retinal and Eye Research.

[bib13] Du J, Linton JD, Hurley JB (2015). Probing metabolism in the intact retina using stable isotope tracers. Methods in Enzymology.

[bib14] Gass JD (1999). Müller cell cone, an overlooked part of the anatomy of the fovea centralis: hypotheses concerning its role in the pathogenesis of macular hole and foveomacualr retinoschisis. Archives of Ophthalmology.

[bib15] Gass JD, Blodi BA (1993). Idiopathic juxtafoveolar retinal telangiectasis. Update of classification and follow-up study. Ophthalmology.

[bib16] Handa JT (2012). How does the macula protect itself from oxidative stress?. Molecular Aspects of Medicine.

[bib17] Klein R, Blodi BA, Meuer SM, Myers CE, Chew EY, Klein BE (2010). The prevalence of macular telangiectasia type 2 in the beaver dam eye study. American Journal of Ophthalmology.

[bib18] Kofuji P, Newman EA (2004). Potassium buffering in the central nervous system. Neuroscience.

[bib19] Lujan BJ, Roorda A, Knighton RW, Carroll J (2011). Revealing Henle's Fiber Layer Using Spectral Domain Optical Coherence Tomography. Investigative Opthalmology & Visual Science.

[bib20] Mori K, Kanno J, Gehlbach PL (2016). Retinochoroidal morphology described by wide-field montage imaging of spectral domain optical coherence tomography. Retina.

[bib21] Newman EA (1996). Acid efflux from retinal glial cells generated by sodium bicarbonate cotransport. The Journal of Neuroscience.

[bib22] Okawa H, Sampath AP, Laughlin SB, Fain GL (2008). ATP consumption by mammalian rod photoreceptors in darkness and in light. Current Biology.

[bib23] Peek R, Verbraak F, Coevoet HM, Kijlstra A (1998). Müller cell-specific autoantibodies in a patient with progressive loss of vision. Investigative Ophthalmology & Visual Science.

[bib24] Perkins GA, Ellisman MH, Fox DA (2004). The structure-function correlates of mammalian rod and cone photoreceptor mitochondria: observations and unanswered questions. Mitochondrion.

[bib25] Perkins GA, Frey TG (2000). Recent structural insight into mitochondria gained by microscopy. Micron.

[bib26] Poitry S, Poitry-Yamate C, Ueberfeld J, MacLeish PR, Tsacopoulos M (2000). Mechanisms of glutamate metabolic signaling in retinal glial (Müller) cells. The Journal of Neuroscience.

[bib27] Poitry-Yamate CL, Poitry S, Tsacopoulos M (1995). Lactate released by Müller glial cells is metabolized by photoreceptors from mammalian retina. The Journal of Neuroscience.

[bib28] Powner MB, Gillies MC, Tretiach M, Scott A, Guymer RH, Hageman GS, Fruttiger M (2010). Perifoveal müller cell depletion in a case of macular telangiectasia type 2. Ophthalmology.

[bib29] Powner MB, Gillies MC, Zhu M, Vevis K, Hunyor AP, Fruttiger M (2013). Loss of Müller's cells and photoreceptors in macular telangiectasia type 2. Ophthalmology.

[bib30] Reichenbach A, Bringmann A (2013). New functions of Müller cells. Glia.

[bib31] Scerri TS, Quaglieri A, Cai C, Zernant J, Matsunami N, Baird L, Scheppke L, Bonelli R, Yannuzzi LA, Friedlander M, Egan CA, Fruttiger M, Leppert M, Allikmets R, Bahlo M, MacTel Project Consortium (2017). Genome-wide analyses identify common variants associated with macular telangiectasia type 2. Nature Genetics.

[bib32] Scott DA, Richardson AD, Filipp FV, Knutzen CA, Chiang GG, Ronai ZA, Osterman AL, Smith JW (2011). Comparative metabolic flux profiling of melanoma cell lines: beyond the warburg effect. The Journal of Biological Chemistry.

[bib33] Sharon D, Blackshaw S, Cepko CL, Dryja TP (2002). Profile of the genes expressed in the human peripheral retina, Macula, and retinal pigment epithelium determined through serial analysis of gene expression (SAGE). PNAS.

[bib34] Song WT, Zhang XY, Xiong SQ, Wen D, Jiang J, Xia XB (2013). Comparison of two methods used to culture and purify rat retinal müller cells. International Journal of Ophthalmology.

[bib35] Too LK, Gracie G, Hasic E, Iwakura JH, Cherepanoff S (2017). Adult human retinal müller glia display distinct peripheral and macular expression of CD117 and CD44 stem cell-associated proteins. Acta Histochemica.

[bib36] Yamada E (1969). Some structural features of the fovea centralis in the human retina. Archives of Ophthalmology.

[bib37] Zhang T, Gillies MC, Madigan MC, Shen W, Du J, Grünert U, Zhou F, Yam M, Zhu L (2018). Disruption of de novo serine synthesis in müller cells induced mitochondrial dysfunction and aggravated oxidative damage. Molecular Neurobiology.

[bib38] Zhu L, Shen W, Lyons B, Wang Y, Zhou F, Gillies MC (2015). Dysregulation of inter-photoreceptor retinoid-binding protein (IRBP) after induced müller cell disruption. Journal of Neurochemistry.

[bib39] Zhu S, Yam M, Wang Y, Linton JD, Grenell A, Hurley JB, Du J (2018). Impact of euthanasia, dissection and postmortem delay on metabolic profile in mouse retina and RPE/choroid. Experimental Eye Research.

